# *Thrips tabaci* Population Genetic Structure and Polyploidy in Relation to Competency as a Vector of *Tomato Spotted Wilt Virus*

**DOI:** 10.1371/journal.pone.0054484

**Published:** 2013-01-24

**Authors:** Alana L. Jacobson, Warren Booth, Edward L. Vargo, George G. Kennedy

**Affiliations:** 1 Department of Entomology, North Carolina State University, Raleigh, North Carolina, United States of America; University of Oxford, United Kingdom

## Abstract

Knowledge of population-level genetic differences can help explain variation among populations of insect vectors in their role in the epidemiology of specific viruses. Variation in competency to transmit *Tomato spotted wilt virus* (TSWV) that exists among populations of *Thrips tabaci* has been associated with the presence of cryptic species that exhibit different modes of reproduction and host ranges. However, recent findings suggest that vector competency of *T. tabaci* at any given location depends on the thrips and virus populations that are present. This study characterizes the population genetic structure of *T. tabaci* collected from four locations in North Carolina and examines the relationship between population genetic structure and variation in TSWV transmission by *T. tabaci*. Mitochondrial COI sequence analysis revealed the presence of two genetically distinct groups with one characterized by thelytokous, parthenogenetic reproduction and the other by arrhenotokous, sexual reproduction. Using a set of 11 microsatellite markers that we developed to investigate *T. tabaci* population genetic structure, we identified 17 clonal groups and found significant genetic structuring among the four NC populations that corresponded to the geographic locations where the populations were collected. Application of microsatellite markers also led to the discovery of polyploidy in this species. All four populations contained tetraploid individuals, and three contained both diploid and tetraploid individuals. Analysis of variation in transmission ofTSWV among isofemale lines initiated with individuals used in this study revealed that ‘clone assignment,’ ‘virus isolate’ and their interaction significantly influenced vector competency. These results highlight the importance of interactions between specific *T. tabaci* clonal types and specific TSWV isolates underlying transmission of TSWV by *T. tabaci*.

## Introduction

Knowledge of population-level genetic differences and clonal diversity in sexual and asexual species has improved our understanding of the role insect vectors have in the epidemiology of specific viruses [1]–[6]. *Thrips tabaci* Lindeman, is a polyphagous insect pest species that is also a vector of two important plant infecting Tospoviruses (Genus: *Tospovirus* Family: Bunyaviridae), *Tomato spotted wilt virus* (TSWV) and *Iris yellow spot virus* (IYSV) [7], [8]. In nature tospoviruses are transmitted exclusively by only 14 of approximately 7,400 described thrips species [9], [10]. Worldwide, *T. tabaci* is the sole vector of IYSV, which is responsible for an estimated U.S. $60–90 million in onion crop losses in the western U.S. [11], and is one of 10 reported thrips vectors of TSWV, estimated to cause over U.S. $1 billion in crop losses annually worldwide [12], [13]. *T. tabaci* was the first species reported to transmit TSWV and was believed to be the primary vector until transmission studies revealed extensive variation in its competence as a vector of TSWV. Brazilian and Canadian populations of *T. tabaci* did not transmit TSWV in transmission assays [14]–[16], whereas in Europe both poor and efficiently transmitting populations are found, sometimes in the same area [17]. Despite this variation *T. tabaci* is the primary vector of TSWV in tobacco and vegetable production systems in Europe and Tasmania, respectively [17]–[21]. In the USA *T. tabaci* is not reported as an important vector of TSWV, however, both poor and efficiently transmitting populations have been observed [22], [23].

Variation in transmission efficiency by *T. tabaci* populations has long been associated with the presence of cryptic species that exhibit different modes of reproduction and host ranges [17], [24], [25], [26], [27]. Zawirska [24] concluded there are two subspecies of *T. tabaci*, one exhibiting arrhenotokous parthenogenesis that is found on tobacco and capable of transmitting TSWV, the other exhibiting thelytokous parthenogenesis that is not found on tobacco and not capable of transmitting TSWV. Two recent studies examining mitochondrial cytochrome oxidase subunit one (mtCOI) sequence variation have provided evidence for at least three genetically distinct groups based on host plant (leek versus tobacco) [26], or reproductive mode (male producing and thelytokous populations) [27]. It remains unknown whether or not ongoing gene flow occurs among these groups. Although inherent differences in vector competency of the different groups and their distributions may play an important role in TSWV disease epidemics, the association of efficient transmitters, inefficient transmitters, and non-transmitters with these groups is not clear. Poor and efficient transmission rates have been observed in both arrhenotokous and thelytokous populations collected from multiple host plants [14]–[17], [22], [23], [28], [29] but no studies have directly compared transmission efficiency among *T. tabaci* individuals in relation to species or population genetic structure. It has been shown that a large amount of variation in transmission of different TSWV isolates exists within purely inbred isofemale lines initiated from individual, parthenogenetic females collected from the same location [22]. Additionally, there is evidence that efficient transmission of TSWV by *T. tabaci* is specific to certain isolate and thrips population combinations, and is more commonly observed when the tested TSWV isolate and thrips combinations were collected from the same location [22]. All of this suggests that defining *T. tabaci* as a TSWV vector on the basis of genetic groupings associated with host plant or reproductive mode alone is not sufficient for understanding the potential of this vector in disease epidemics.

The objectives of this study were to characterize the population genetic structure of *T. tabaci* collected from four different locations in North Carolina using mtCOI sequences and microsatellite markers, and to examine whether there is a relationship between genetic structure and variation in TSWV transmission by *T. tabaci*. Isofemale lines established from parthenogenetically reproducing, field-collected females that were previously characterized for both reproductive mode type and their ability to transmit several TSWV isolates were used in this study [22]. In addition to characterizing genetic structure, clonal assignments based on allele similarities of microsatellite loci were incorporated into statistical models describing the relationships between TSWV isolate and thrips to test for the significance of thrips clones in determining transmission efficiency. Our results show the presence of two genetically distinct groups based on reproductive mode, that populations of *T. tabaci* are geographically structured across the sampled region, and that the effect of clonal assignment in statistical models was a significant factor used to estimate the probability of TSWV transmission by *T. tabaci*.

## Materials and Methods

### Ethics statement

No specific permits were required for the described field studies. No permission was required to collect insects or plant material from Jackson Springs or Mills River collection sites. These reside on university research farms (with whom we are affiliated) or unregulated public lands. Verbal permission was obtained from private landowners for the Apex and Faison collections sites, respectively. The field studies did not involve regulated, endangered, or protected species.

### Insect collection

**Figure 1:**
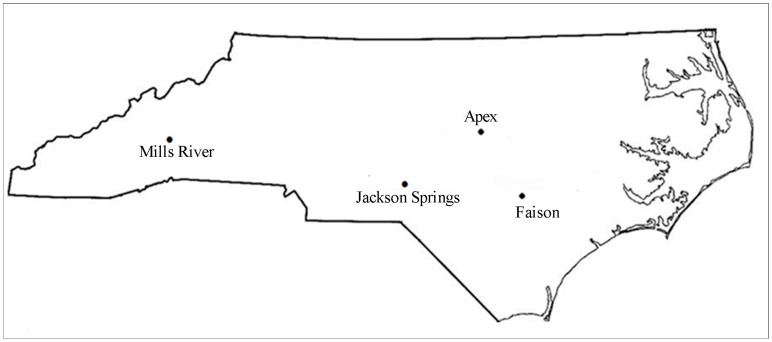
Map of North Carolina collection sites for the four *Thrips tabaci* Populations.

*Thrips tabaci* individuals were collected from cultivated and weed plant hosts at different locations in North Carolina during May and June of 2010 (Table 1, Figure 1). These individuals are the same reported in Jacobson and Kennedy [22]; however, only individuals from the four field sites at which at least eight individuals were collected were included in this study (Table 1). Field collected individuals were kept separate, and parthenogenetic females were used to establish 37 isofemale lines. The offspring of each of these females were then divided into three groups. One group of virgin females was used to characterize reproductive mode based on the sex of their offspring: parthenogenetically, thelytokous individuals produce only females, arrhenotokous individuals produce only males, and deuterotokous individuals produce males and females. Voucher specimens of isofemale lines were deposited in the North Carolina State University Insect Museum. The second subgroup was used in TSWV transmission assays [22]. The third group was stored at −20°C in 95% ethanol for population genetic analysis. The thrips used in this study included a subsample of individuals from parthenogenetic, isofemale lines previously characterized for their ability to transmit 2–4 isolates of TSWV collected from NC (Table 1) [22]. The proportion of isofemale line progeny that transmitted TSWV to indicator leaf discs for this subgroup of isofemale lines ranged from 0–0.55. The majority of these individuals were thelytokous but high transmission rates were observed in both arrhenotokous and thelytokous isofemale lines. All isofemale lines were able to transmit at least one of the TSWV isolates. Isofemale lines were reared in isolation from each other and no more than two generations of parthenogenetic reproduction separated the individuals used in the transmission study and the individuals used in the population genetic study.

**Table 1:** *Thrips tabaci* samples collection information.

Isoline	Location	Host Plant	RM^1^	Ploidy^2^	Clone^2^	mtCOI^3^	GenBank Accession#^3^
Apex-1	Apex, NC: 35.718306N, −78.926981W	*Allium cepa*	T	4	1	1	JX403008
Apex-2	Apex, NC: 35.718306N, −78.926981W	*Allium cepa*	T	4	2	—	—
Apex-4*	Apex, NC: 35.718306N, −78.926981W	*Allium cepa*	T	4	4	—	—
Apex-6	Apex, NC: 35.718306N, −78.926981W	*Allium cepa*	T	4	2	—	—
Apex-10	Apex, NC: 35.718306N, −78.926981W	*Allium cepa*	T	4	2	—	—
Apex-11	Apex, NC: 35.718306N, −78.926981W	*Allium cepa*	T	4	2	—	—
Apex-14	Apex, NC: 35.718306N, −78.926981W	*Allium cepa*	T	4	3	4	JX403011
Apex-16	Apex, NC: 35.718306N, −78.926981W	*Allium cepa*	T	4	2	—	—
Cot1-2*	Faison, NC: 35.1212N, −78.1582W	*Raphanus raphanistrum*	T	4	1	1	—
Cot1-3	Faison, NC: 35.1212N, −78.1582W	*Raphanus raphanistrum*	T	4	1	1	—
Cot1-4	Faison, NC: 35.1212N, −78.1582W	*Raphanus raphanistrum*	T	4	1	—	—
Cot1-5	Faison, NC: 35.1212N, −78.1582W	*Raphanus raphanistrum*	T	2	1	—	—
Cot1-6	Faison, NC: 35.1212N, −78.1582W	*Allium cepa*	T	4	1	1	—
Cot1-8*	Faison, NC: 35.1212N, −78.1582W	*Brassica oleracea*	T	4	1	1	—
Cot1-10	Faison, NC: 35.1212N, −78.1582W	*Brassica oleracea*	T	4	1	1	—
Cot2-1*	Faison, NC: 35.1223N, −78.131W	*Raphanus raphanistrum*	T	4	1	1	—
Cot2-2 *	Faison, NC: 35.1223N, −78.131W	*Allium spp.*	T	2	1	1	—
Cot2-4*	Faison, NC: 35.1223N, −78.131W	*Allium cepa*	T	2	1	—	—
MHC1*	Mills River, NC: 35.4292N, −82.5614W	*Allium cepa*	A	1	17	6	JX403013
MHC2*	Mills River, NC: 35.4292N, −82.5614W	*Allium cepa*	A	2	16	6	—
MHC7	Mills River, NC: 35.4292N, −82.5614W	*Allium cepa*	A	2	6	6	—
MHC8	Mills River, NC: 35.4292N, −82.5614W	*Allium cepa*	A	2	7	—	—
MHC9	Mills River, NC: 35.4292N, −82.5614W	*Allium cepa*	A	4	8	6	—
MHC11	Mills River, NC: 35.4292N, −82.5614W	*Allium cepa*	A	2	12	7	JX403014
MHC19	Mills River, NC: 35.4292N, −82.5614W	*Allium cepa*	A	2	8	6	—
MHC33	Mills River, NC: 35.4292N, −82.5614W	*Allium cepa*	A	2	13	—	—
MHC39	Mills River, NC: 35.4292N, −82.5614W	*Allium cepa*		—	—	6	—
MHC41	Mills River, NC: 35.4292N, −82.5614W	*Allium cepa*	A	1	5	6	—
MHC53	Mills River, NC: 35.4292N, −82.5614W	*Allium cepa*	A	4	15	—	—
SH2*	Jackson Springs, NC: 35.1938N, −79.6848W	*Secale cereale*	T	4	1	—	—
SH10	Jackson Springs, NC: 35.1938N, −79.6848W	*Brassica spp.*	T	4	9	—	—
SH28	Jackson Springs, NC: 35.1938N, −79.6848W	*Brassica spp.*	T	4	10	—	—
SH30	Jackson Springs, NC: 35.1938N, −79.6848W	*Brassica spp.*	T	2	1	—	—
SH45	Jackson Springs, NC: 35.1938N, −79.6848W	*Brassica spp.*	T	2	1	1	—
SH61	Jackson Springs, NC: 35.1938N, −79.6848W	*Allium spp.*	T	—		3	JX403010
SH63*	Jackson Springs, NC: 35.1921N, −79.6868W	*Allium spp.*	T	4	14	1	—
SH68*	Jackson Springs, NC: 35.1799N, −79.6749W	*Allium spp.*	T	4	9	5	JX403012
SH69	Jackson Springs, NC: 35.193N, −79.6819W	*Raphanus sativus* var. *niger*	T	—	—	2	JX403009
SH72*	Jackson Springs, NC: 35.193N, −79.6819W	*Raphanus sativus* var. *niger*	T	2	9	—	—
SH75	Jackson Springs, NC: 35.1938N, −79.6848W	*Brassica spp.*	T	4	10	—	—

1 Reproductive Mode.

2 Based on microsatellite marker data.

3 mtCOI haplotypes.

* characterized for transmission efficiency in [22].

### DNA extraction and molecular screening

The sex of thrips was verified visually under a compound light microscope and recorded for each individual before DNA extraction. Total genomic DNA was extracted from individual thrips using the DNeasy® Blood and Tissue Kit (QIAGEN, Valencia, CA) according to manufacturer's instructions. DNA was eluted in 30 µl of AE buffer.

### Mitochondrial DNA sequencing and analysis

From the sampled populations, a subset of individuals were amplified for a 629-bp fragment of the mitochondrial cytochrome oxidase I (COI) gene using primers LepF1 (5′-ATTCAACCAATCATAAAGATATTGG-3′) and LepR1 (5′-TAAACTTCTGGATGTCCAAAAAATCA-3′) [30], [31]. Polymerase chain reactions (PCRs) were performed in 25 µl volumes containing: 1× PCR buffer, 2 mM MgCl_2_, 100 µM dNTPs, 3 pM of each primer, 0.5 U Taq DNA polymerase (Bioline, Taunton, MA), 2 µl of DNA, and ddH_2_O to 25 µl. PCR cycling conditions were comprised of an initial denaturation stage of 1 min at 94°C, followed by 35 cycles each consisting of 1 min at 94°C, 1.5 min at 45°C, and 1.5 min at 72°C, with a final elongation step at 72°C for 5 minutes, carried out using a PTC-200 thermal cycler (MJ Research Inc.). PCR products were visualized on a 2.5% agarose gel to confirm samples contained a single band, and 5 µl of PCR product was subsequently purified using the ExoSAP-IT PCR purification kit (USB Corporation, OH, USA) and bidirectionally sequenced following the methodology outlined in Copren et al. [32].

Mitochondrial COI sequence alignments were performed using the Vector NTI Advance 10 program (Invitrogen, Carlsbad, CA). Phylogenetic relationships were examined using Molecular Evolutionary Genetics Analysis (MEGA), Version 4 [33]. Neighbor-joining (NJ), UPGMA, and Minimum Evolution analyses in which all characters were equally weighted and 10,000 bootstrap replicated were conducted. Minimum spanning networks were also constructed to examine the evolutionary relationship between the mtDNA sequences using the program TCS 1.21 [34].

### Microsatellite development and analysis

From five *T. tabaci* specimens, selected from geographically distant sampling locations, DNA was extracted using the DNEasy Blood & Tissue kit (Qiagen, Valencia, CA). DNA quality and concentration from each specimen were determined using the BioSpec-nano spectrophotometer (Shimadzu Scientific Instruments, Columbia, MD). Pooled DNA from the five specimens was then subjected to shotgun sequencing using the Roche 454 Genome Sequencer FLX (Roche Applied Science, Penzberg, Germany) with the Titanium Sequencing kit XLR 70, performed at the Genomic Sequencing Laboratory located at the North Carolina State University. Sequencing was performed on a 1/16 GS-FLX PTP.

A total of 97,611 reads were obtained with an average read length of 354 bp and a total of 34,514,909 bp. Using MSATCOMMANDER version 0.8.2 [35], all unassembled sequences were screened for di-, tri-, and tetranucleotides using default settings within the program. Primers were designed using the PRIMER3 software [36], implemented within the MSATCOMMANDER program, and tagged with a 19 base pair (bp) M13 forward label (CACGACGTTGTAAAACGAC). Amplification products were chosen to be within a 100 to 400 bp range (including M13 tag), with an optimal annealing temperature of 59°C (range 57°C–63°C), an optimal GC content of ∼50%, low levels of self- or pair-complementarity, and a maximum stability of 8.0 [35]. Following the removal of duplicate sequences, a total of 879 sequences were found to contain tandem repeats within the desired criteria with sufficient flanking region for primer design: 352 di-, 304 tri-, and 222 tetra-nucleotide microsatellites with at least 10, 5, and 5 repeats, respectively. Of these, 52 primer pairs were tested with 11 selected for microsatellite analysis (Table 2).

**Table 2:** Microsatellite loci primer information.

Locus	Primer sequences (5′ - 3′)	Repeat motif	µM each primer	Allele size range (bp)	GenBank Accession #
*T.tab*-6	F: CACGCAAAACACTCTCTCCA	(ACAG)^11^	0.11	172–261	JX402997
	R: AGTGGCGTCTGTGTTGAGAA		0.11		
*T.tab*-20	F: ACCGGAAGCTTTCAAATCG	(AGCC)^9^	0.11	117–257	JX402998
	R: AATAAACCGTCGCGGAGACT		0.11		
*T.tab*-24	F: GTAGAGCAGCACCGATAGGG	(AAC)^10^	0.10	294–320	JX402999
	R: CAGCCAGGACAACAGAGTGA		0.10		
*T.tab*-27	F: AAGGTCAGGCATTGCGTTAT	(AAC)^8^	0.10	312–343	JX403000
	R: TACAAAGCGAGGACTCAGCA		0.10		
*T.tab*-29	F: TTCATTTTGCAGTGGCAACTAT	(AAT)^13^	0.10	273–296	JX403001
	R: GAGTCTGCGTCGTGGATATG		0.10		
*T.tab*-33	F: TCGTGGCATGACTCAAACG	(AC)^12^	0.10	150–184	JX403002
	R: CCTCGGAACAAGGAGCCAG		0.10		
*T.tab*-34	F: TTTGCTGTCCCTCGAAGCG	(AC)^26^	0.10	139–168	JX403003
	R: CGATTCCATGTTTGTCTAAGAGTCC	0.10			
*T.tab*-43	F: GCTCCCGCACCAGAGAATTAC	(AC)^14^	0.10	143–171	JX403004
	R: ACGTTCCTTTGGAGTTCCAGC		0.10		
*T.tab*-47	F: TTCCTCGCGTGCCCTATG	(AC)^15^	0.15	212–234	JX403005
	R: GTCGTGTAGCTGGAAGTGC		0.15		
*T.tab*-48	TCGAACGGCTGGTGTGAAG	(AC)^16^	0.10	192–227	JX403006
	GCGACCATTCGCGGTTC		0.10		
*T.tab*-49	F: CGGACATGCGACATTCACC	(AC)^17^	0.10	276–304	JX403007
	R: CGGAATTCGGAGCGAGCC		0.10		

Primer pairs were optimized using 10 individual *T. tabaci*. PCRs were carried out in 12 µl total volumes, each containing 1× PCR buffer, 1.75 mM MgCl_2_, 100 mM dNTPs, ∼20 ng DNA template, 0.5 U Apex *Taq* DNA polymerase (Genesee Scientific, San Diego, CA), and ddH_2_O to 12 µl. Primer concentration varied between 0.10 and 0.15 µM (Table 2) with the forward primer end-labeled with an M13F-29/IRD700 or 800 IRDye tag (Li-Cor Inc., Lincoln, NE). PCR cycling conditions were comprised of an initial denaturation stage of 3 min at 95°C, followed by 29 cycles consisting of 30 s denaturation at 95°C, 30 s at optimal annealing temperature of 59°C, and 30 s extension at 72°C. Following PCR, 5 µl of stop solution (95% formamide, 20 mm EDTA, bromophenol blue) was added to each reaction. Reactions were subsequently denatured at 95°C for 4 min prior to loading onto a 25 cm 6% polyacrylamide gel, using either 50–350 bp or 50–700 bp IRDye standards (Li-Cor Inc., Lincoln, NE) for accurate product sizing. Results were analyzed using the GeneProfiler software (Scanalytics, Inc., BD Biosciences Bioimaging, Rockville, MD).

Clonality and polyploidy of NC *T. tabaci* populations had to be considered during microsatellite analyses. The *T. tabaci* individuals used in this study exhibited arrhenotoky and thelytoky, and females from NC were shown to be both diploid and polyploid during microsatellite marker development for this project [37]. Ploidy could not be determined for the specific individuals included in this study because storage of the thrips in 95% ethanol was not compatible with flow cytometry methods used for ploidy determination [37]. However, results from a flow cytometry and microsatellite marker analysis of other individuals taken from the isofemale lines maintained in the laboratory suggest that these populations are tetraploid based on the observed genome sizes of the polyploids, and the maximum number of microsatellite alleles per locus in individuals across North Carolina [37]. Additionally, it was shown that the maximum number of alleles observed with the microsatellite markers used in this study did provide reliable estimates of ploidy [37]. Therefore, ploidy estimates were generated based on maximum allele copy number. Female individuals exhibiting a maximum of two alleles across all loci were characterized as diploid and individuals with more than two bands at any locus were characterized as tetraploid [37]. Males included in this study had a maximum of one allele per locus.

The R program Polysat [38] was used for analysis because it can handle samples of mixed ploidy, provides methods to estimate allele copy number in partial heterozygote polyploids, and calculates clonal diversity statistics. Pairwise genetic Bruvo distances were then calculated using the method described by Bruvo et al. [39]. This pairwise genetic distance matrix was then used to perform a principle component analysis (PCA) to examine population structuring by plotting the first two principle components. Groups of asexually related individuals were assigned to clonal groups using the Bruvo distance matrix. A distance measure of 0.2 was used for these assignments based on the histogram of the distribution of genetic Bruvo distances. Allele frequencies were then estimated using the simpleFreq function and pairwise *F*_ST_ values calculated based on the methods of Nei [40]. Isolation by distance was also calculated based on pairwise *F*_ST_ and distance matrices in Genepop using the isolde suboption with a Mantel's test with 1000 permutations, log transformations of distance and *F*/(*F*-1)-statistics [41].

### Population structure and TSWV transmission

A previous TSWV transmission study conducted with individuals from the same isofemale line showed that isolate, isofemale line and their interaction were statistically significant effects describing the probability of successful transmission of TSWV [22]. Additionally, higher transmission rates were seen among sympatric TSWV isolate-isofemale line pairings (collected from the same location) versus allopatric TSWV isolate-isofemale line pairings (collected from different locations). These results indicate that both viral and thrips genetic components underlie successful transmission of TSWV by *T. tabaci*, and suggest that local adaptation may be occurring between TSWV isolates and thrips. To further investigate the virus-vector relationship between TSWV isolates and *T. tabaci*, the analysis of TSWV transmission was re-run using 12 isofemale lines that came from one of the four populations used in the genetic analysis for which both clonal assignments and TSWV transmission data in individuals were available, along with two additional isofemale lines, one from Kinston, NC and one from Cove City, NC, for which transmission and microsatellite clone assignment information was available. Not enough transmission data were available for the Mills River isofemale lines to include them in this analysis. Models were run using Proc Glimmix (SAS Institute, Cary, NC). The mtCOI haplotypes were not included in this analysis because the small number of arrhenotokous individuals available did not permit a robust comparison.

## Results

### Genetic analysis

**Figure 2:**
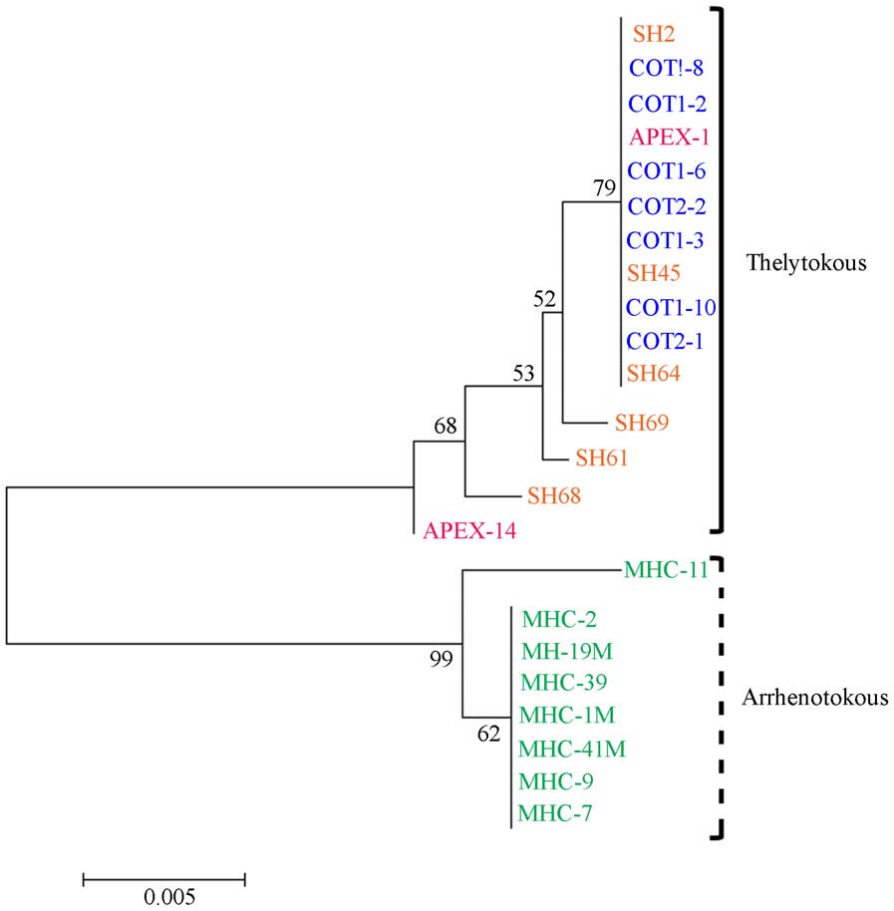
Phylogenetic tree constructed from mitochondrial DNA sequences from four North Carolina *T. tabaci* populations using the Neighbor-joining method (Mills River – green; Apex – pink; Faison – blue; Jackson Springs – orange). Nonparametric bootstrap values are shown above branches.

NJ, UPGMA, and Minimum Evolution phylogenetic methods employed in MEGA produced identical tree topologies (Figure 2). The individuals in these populations were subdivided into two groups that corresponded with their reproductive mode. Group one was comprised of all of the thelytokous individuals and group two was comprised of the arrhenotokous individuals. Within group sequence divergence estimates for groups one and two were 0.23% (±0.10%) and 0.16% (±0.08%), respectively. Overall mean sequence divergence estimates among samples were 1.74% (±0.36%), whereas between group mean sequence divergence was 3.42% (±0.72%).

**Figure 3:**
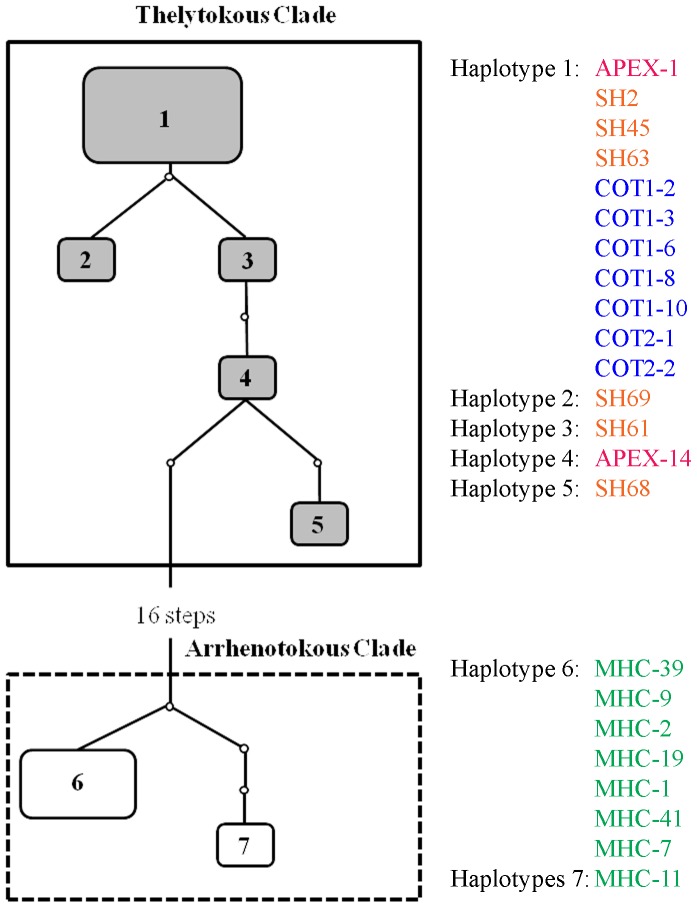
Minimum spanning network calculated with mitochondrial COI sequences using TCS 1.2 1 (Mills River – green; Apex – pink; Faison – blue; Jackson Springs – orange). Haplotypes of thelytokous and arrhenotokous North Carolina *Thrips tabaci* are shown in grey and white, respectively. Boxes grouping haplotypes indicate networks identified with 90% probability of parsimony.

Minimum spanning networks generated with TCS produced two main subgroups comprised of thelytokous and arrhenotokous individuals, respectively (Figure 3). The thelytokous individuals cluster together and are connected to each other by a maximum of six mutational steps. The arrhenotokous individuals also cluster together and are connected to each other by a maximum of 4 mutational steps. Arrhenotokous and thelytokous individuals, however, are separated by a minimum of 18 mutational steps, which supports the results from the phylogenetic analysis, and the previous analysis of Brunner et al. [26] that shows genetic variation sufficiently large to suggest these groups represent different subspecies or biotypes.

### Population genetic analysis

All of the individuals collected from Apex, Jackson Springs, and Faison exhibited thelytokous parthenogenesis, and all of the individuals from Mills River exhibited arrhenotokous parthenogenesis. Both diploid and tetraploid females were found in Faison, Jackson Springs and Mills River collection sites, whereas only tetraploids were collected from Apex. Males from Mills River included in the study appeared to be haploid based on the maximum number of alleles observed at each microsatellite locus, however, ploidy has not been formally examined for male *T. tabaci* [37]. Mean allelic richness was four alleles/locus, and ranged from three-six when analyzed per locus and per population. A total of 17 clonal groups were identified from these four populations (Table 1). No arrhenotokous individual belonged to the same clonal group as a thelytokous individual, and both diploid and tetraploid individuals were found in the same clonal groups.

**Figure 4:**
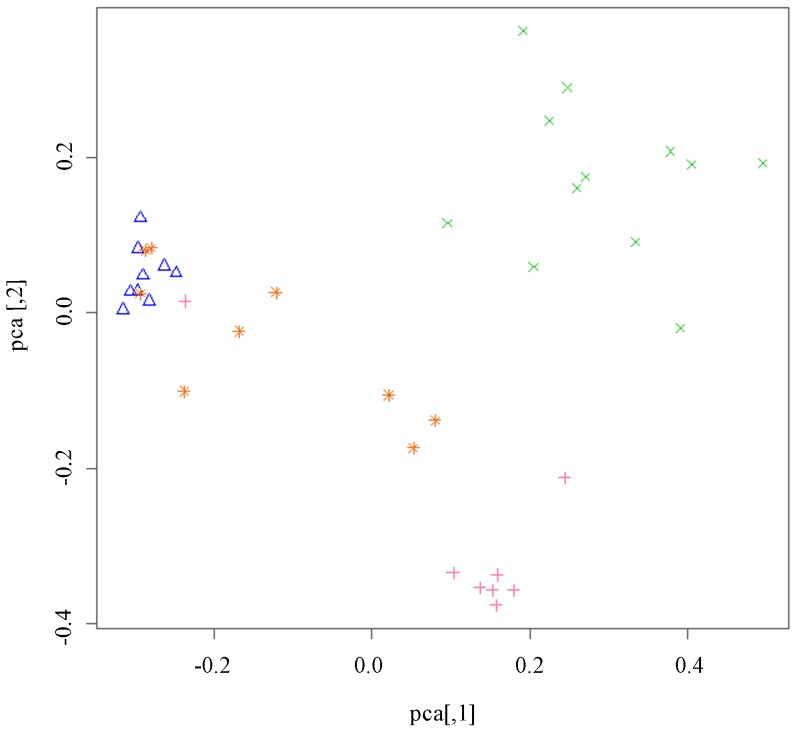
Principle component analysis of the two primary components of pairwise genetic distances calculated for four North Carolina populations of *T. tabaci* at 12 microsatellite loci using distance methods of Bruvo et al. (2004) (Mills River – green x; Apex – pink +; Faison – blue triangle; Jackson Springs – orange asterisk).

**Figure 5:**
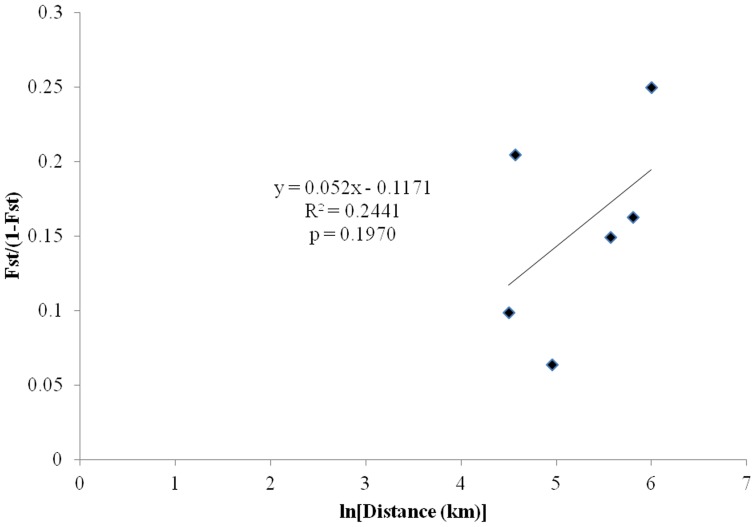
Scatter plot of transformed pairwise *F*_ST_ values and pairwise geographic distances between North Carolina populations of *T. tabaci* with regression line, R^2^ value and *P* value from a Mantel's test for isolation by distance.

The principle component analysis conducted on the first two principle components of the genetic distance matrix calculated with the Bruvo distance measure (Figure 4), pairwise *F*_ST_ (Table 3) and clonal assignments (Table 1) all suggest that populations are geographically structured across NC and that migration of individuals occurs among some of the locations. Individuals from Mills River form 10 unique clonal groups that cluster together in the PCA analysis, and appear to be genetically isolated from the other populations. Three different clonal groups were identified from Apex, and form a tight cluster with each other and separate from the other populations, with the exception of one individual. Individuals from Faison, four individuals from Jackson Springs, and one individual from Apex cluster as a group and belong to the same clonal assignment. The remaining individuals from Jackson Springs form a loose cluster comprised of individuals from three clonal groups. Pairwise *F*_ST_ values are also higher between populations that do not cluster close together in the PCA analysis, which supports the overall pattern of geographic structuring (Table 3). Although neighboring populations appear to be more genetically similar to each other than geographically distant ones in the PCA, the test for isolation by distance was not significant (r^2^ = 0.2441, *P* = 0.1970) (Figure 5).

**Table 3:** Pairwise *F*_ST_ and pairwise distances (km) between four NC populations of *T. tabaci*.*

Pairwise *F*_ST_				
	Apex	Faison	Mills River	Jackson Springs
Apex	0.00			
Faison	0.17	0.00		
Mills River	0.14	0.20	0.00	
Jackson Springs	0.09	0.06	0.13	0.00

Pairwise Distance (km)				
	Apex	Faison	Mills River	Jackson Springs
Apex	0.00			
Faison	96.00	0.00		
Mills River	332.00	402.00	0.00	
Jackson Springs	90.00	142.00	261.00	0.00

* Reproductive modes: Thelytokous – Apex, Faison, Jackson Springs; Arrhenotokous – Mills River.

### Population structure and TSWV transmission

The probability of TSWV transmission based on the variables ‘virus isolate‘, ‘isofemale line’ and ‘sympatry’ [22] was re-analyzed to further investigate the role of thrips genetic components in the transmission of TSWV by including genetic variables for clonal group calculated from microsatellite marker data. First, the statistical models used by Jacobson and Kennedy [22] were re-run on the subsample of isofemale lines that were included in the population genetic analysis. These analyses showed that the main effect of ‘isofemale line’ (*P* = 0.0552) was not significant, but ‘virus isolate’ (*P*<0.0001) and ‘isofemale line×virus isolate’ interaction (*P* = 0.0070) were statistically significant with this subgroup. When the variable ‘sympatry’, replaced the interaction term in this model to investigate whether this interaction was significant among virus isolates and thrips isofemale lines that occur in the same location, ‘virus isolate’ (*P*<0.0001) and ‘isofemale line’ (*P*<0.0001) were significant, but ‘sympatry’ (*P* = 0.4436) was not significant.

Next, these models were run using genetic clonal groups, identified from microsatellite marker analysis, as the variable to describe the thrips isofemale lines. A comparison of transmission efficiency based on mtCOI haplotype could not be conducted due to the small sample sizes of transmission phenotypes available for arrhenotokous haplotypes. When ‘isofemale line’ was replaced with ‘clone assignment’ as the variable to model thrips transmission, ‘clone assignment’ (*P*<0.0001) and ‘virus isolate’ (*P*<0.0001) were significant main effects, but ‘sympatry’ was not (*P* = 0.9577). When ‘clone assignment’ (*P*<0.0001), ‘virus isolate’ (*P*<0.0001) and their interaction term (*p* = 0.0399) were included in the model, however, all three terms were statistically significant. These results highlight the importance of interactions between specific *T. tabaci* clonal types and specific TSWV isolates in determining transmission of TSWV by *T. tabaci*.

## Discussion

The first objective of this study was to characterize the population genetic structure of *T. tabaci* collected from four different locations in NC using mtCOI sequences and microsatellite markers. The results of the mtCOI sequence analysis support previous evidence for the existence of genetically distinct groups within this species [26], [27], and show that two genetically distinct groups are present in NC (Figure 2). The minimum spanning haplotype networks also show strong differentiation between reproductive types (Figure 3) and support the results of the phylogenetic analysis.

This is the first study to examine the population genetic structuring of *T. tabaci* populations using microsatellite markers. Microsatellite markers developed here revealed genetic structure among the four NC populations that corresponded to both the geographic locations where the populations were collected, and the grouping of individuals observed in the phylogenetic analysis conducted with mtCOI sequences. Pairwise *F*_ST_ values ranged from 0.06–0.20 indicating moderate genetic differentiation between all populations (Table 3). The PCA and clonal assignments suggest that arrhenotokous and thelytokous populations are reproductively isolated from each other because arrhenotokous and thelytokous populations do not cluster together or share clonal types. Isolation by distance was not significant among these populations (Figure 5), however this could be due to a small population sample size. Results suggesting reproductive isolation may also be confounded by distance and geographic barriers. North Carolina is comprised of three geographic regions that vary in climate and topography; the mountains, the piedmont and the coastal region. Clonal admixture only occurred among the three thelytokous populations that were collected in the central piedmont and coastal region. These three populations were separated by 142 km or less, suggesting that dispersal occurs at this distance. The arrhenotokous population was collected in a field located in the Appalachian Mountains, and was therefore separated from the thelytokous populations by two potential geographic barriers to dispersal and gene flow: the mountains, and the 261–402 km distance that separate them. Additional studies of population structure should be conducted where thelytokous and arrhenotokous individuals coexist to further investigate breeding structure of local populations and the potential for interbreeding between populations with different reproductive modes.

The discovery of polyploidy in *T. tabaci* populations during microsatellite marker development was unexpected, and is the first report of polyploidy in Thysanoptera [37]. NC populations of *T. tabaci* appeared to be diploid and tetraploid based on genome size estimates for multiple individuals. Because we did not expect populations to be polyploid our sample collection, storage, and processing methodology only allowed for the determination of ploidy and allele dosage in these populations using allele frequency based estimation methods. Based on these methods, all populations sampled contained tetraploid individuals, and three of the populations were comprised of both diploid and tetraploid individuals. Polyploidy represents a significant barrier to the establishment of, and gene flow among populations due to the elevated likelihood of genetic incompatibility amongst individuals of different ploidy level [42]–[44]. This may be circumvented through the establishment or transition to asexual/parthenogenetic reproduction [44]. Following the establishment of a facultatively parthenogenetic phase (asexual reproduction by a sexually reproducing species), populations may then be established through inbreeding. Outbreeding with diploids, in contrast, yielding triploid offspring, is considered a reproductive dead-end due to the increased likelihood of reduced fertility [44]. Polyploidy, and thus clonality have been thought to contribute to the successful spread and establishment of individuals into marginal habitats. Research examining the effects of both clonality and polyploidy among arthropods showed that in some species the abundance and distribution of clones is better described when geographic patterns of polyploidy are examined alone, or with distribution of parthenogens [45]. Thelytokous *T. tabaci* are more common worldwide; males and arrhenotokous populations are rarely reported in the literature, and nothing is known about the abundance and distribution of polyploids. Although this analysis does not suggest that genetic structuring occurs as a result of polyploidy in the NC populations sampled, tetraploidy was almost twice as common in these populations, and may be contributing to the successful establishment of clones in some areas. Specific effects of polyploidy have been reviewed recently in a series of publications ([46]–[52], and references therein), and include discussions on the production and maintenance of polyploids, genomic duplication and reduction events, genomic structuring, gene expression, gene dosage, changes in physiology, development, and fitness, breeding structure, genetic diversity, heterozygosity and inbreeding. More research is needed to address how polyploidy influences these traits in *T. tabaci*, especially with regard to the origin and presence of polyploidy, the relative distributions of diploid and polyploid individuals, and how polyploidy influences population structure in arrhenotokous populations. Another important area of research will be whether or not elevated gene copies are maintained and expressed, especially in relation to traits that may influence acquisition, infection and transmission of viruses, or genes related to insecticide metabolism or target sites whose elevated expression has been related to insecticide resistance in hemipteran and dipteran species [53].

The premise of this study to relate *T. tabaci* population structure to transmission efficiency of TSWV was born from observations that thrips and TSWV isolate, when grouped by location, were both significant explanatory variables in accounting for variation in the probability of TSWV transmission, and that, on average, transmission rates were higher among sympatric TSWV isolate-thrips pairings [22]. Investigating the probability of TSWV transmission by *T. tabaci* in relation to a specific ‘clonal assignment’ variable that uses the genetics of individual thrips, rather than using general population definitions such as ‘isofemale line’ that groups thrips transmission phenotypes based on where they were collected, revealed different, yet related, thrips tospovirus interactions. These interactions offer insights into the localized importance of this species as a vector of TSWV and highlight the role of genetic variation in isolate-specific vector competency among individual thrips within and among populations of *T. tabaci* in determining this species role in the epidemiology of TSWV over a broad range of spatial dimensions. While local adaptation between TSWV and *T. tabaci*, suggested by the significance of sympatry in earlier models, contributes to observed variation in vector competency among *T. tabaci* populations, ultimately, the interaction of specific thrips genotypes with specific virus isolates underlies transmission of TSWV by *T. tabaci*. Therefore, when assessing the importance of *T. tabaci* as a vector of TSWV, efficient transmission more likely depends on specific genotypic interactions between thrips clonal groups and virus isolates than on cryptic species group alone. However, cryptic species group, reproductive mode, and other life history traits affecting the fitness of these individuals will influence the abundance and distribution of efficient vectors through time and space. A better understanding of thrips population biology and defining the abundance and distribution of cryptic species and clonal groups over time would augment our understanding of thrips population dynamics and help to explain patterns of intra-and inter-population variation of economically important traits including vector competency.
